# Modulating the serine metabolism in human differentiated astrocytes: an integrated multi omics approach

**DOI:** 10.3389/fncel.2025.1616911

**Published:** 2025-07-21

**Authors:** Farida Tripodi, Elisa Maffioli, Silvia Sacchi, Valentina Rabattoni, Zoraide Motta, Claudia Bearzi, Gabriella Tedeschi, Loredano Pollegioni, Paola Coccetti

**Affiliations:** ^1^Department of Biotechnology and Biosciences, University of Milano-Bicocca, Milan, Italy; ^2^Department of Veterinary Medicine and Animal Science (DIVAS), University of Milan, Lodi, Italy; ^3^The Protein Factory 2.0 Laboratory, Department of Biotechnology and Life Sciences, University of Insubria, Varese, Italy; ^4^Institute for Biomedical Technologies, National Research Council of Italy (ITB-CNR), Milan, Italy; ^5^CIMAINA, University of Milan, Milan, Italy

**Keywords:** phosphorylated pathway, human differentiated astrocytes, serinosome, metabolism, proteomics, metabolomics

## Abstract

**Introduction:**

Astrocytes are the major source of L-serine (L-Ser) in the brain: the glycolytic intermediate D-3-phosphoglycerate is converted into L-Ser through the phosphorylated pathway (PP) made up of three enzymes, phosphoglycerate dehydrogenase (PHGDH), phosphoserine aminotransferase (PSAT) and phosphoserine phosphatase (PSP), recently proposed to generate a metabolic assembly named serinosome. In the central nervous system, L-Ser is used for a number of functions, including the synthesis of glycine (Gly) and D-serine (D-Ser), the two key NMDAR co-agonists.

**Methods:**

Here, we used iPSC-derived human astrocytes as a cellular model to evaluate the impact on cell metabolism of the overexpression of each of the three enzymes of the PP as GFP-tagged proteins.

**Results:**

The subcellular cytosolic localization of PP enzymes remains unchanged compared to endogenous proteins, while the complex formation is increased in all cases. Notably, among the factors involved, the overexpression of PHGDH appears to play a pivotal role in promoting the serinosome assembly and/or stabilization, highlighting the critical importance of this multi-domain protein. Particularly, the overexpression of each enzyme of the PP alters the cellular metabolism in a specific way. The L-Ser and Gly levels increase more in PHGDH overexpressing cells, in agreement with the known kinetics of the PP. A consistent increase in the TCA cycle, as well as in mitochondrial activities, serine-glycine-one carbon pathway, asparagine, arginine, purine and pyrimidines metabolism is also observed.

**Discussion:**

Peculiar alterations are observed when each enzyme of the PP is overexpressed, strongly supporting the use of human iPSC-derived astrocytes overexpressing the PP pathway enzymes as a valuable cellular model for understanding how Ser glial metabolism occurs in a non-tumor system under both physiological and pathological conditions.

## 1 Introduction

Neurons and glia (astrocytes, oligodendroglia, radial glia, and microglia) are the neural cells of the central nervous system (CNS). Astrocytes, the most abundant type of glial cells in the brain, play an essential role in neurotransmission as an integral part of tripartite synapses. At the synapse, astrocytes are involved in the regulation of ionic balance, neurotransmitter clearance and signaling molecules release, and in brain they are also involved in the regulation of blood flow, ion and water homeostasis, and immune functions (Allen, 2014; Sibille et al., 2014; Khakh and Deneen, 2019; Ziff et al., 2022). Astrocytes are also main regulators of brain metabolism: bioenergetic and antioxidant defenses in the brain are coupled between neurons and astrocytes. E.g., oxidative and nitrosative stress underlie the pathogenesis of a broad range of human diseases, in particular, neurodegenerative disorders such as Alzheimer’s disease (AD), Parkinson’s disease (PD), and stroke (Kim et al., 2019). This bidirectional communication between glia and neuronal elements led to the concept of the tripartite synapse, resulting from the interaction of the presynaptic terminal, the postsynaptic cell and the surrounding astrocyte. Notably, astrocytes display calcium-based excitability and are able to act as sensors and modulators of synaptic transmission and plasticity: calcium elevation responding to synaptically released neurotransmitters stimulates the release of numerous molecules (often named gliotransmitters) which, acting on neuronal receptors, actively participate in synapse formation, function, elimination, plasticity, differentiation, neuronal growth and survival (Chung et al., 2013; Verkhratsky et al., 2016; Tiwari et al., 2018). On this side, D-serine (D-Ser) has gained significant attention: D-Ser acts as a co-agonist of N-methyl-D-aspartate receptors (NMDAR), by binding specifically to GluN1/GluN2A subunit-containing NMDARs (Mothet et al., 2000; Yang et al., 2003; Martineau et al., 2014; Coyle et al., 2020). It is synthesized from L-serine (L-Ser) by the enzyme serine racemase (SR): neurons are the primary producers of D-Ser, although SR expression and D-Ser production has been detected in a subpopulation of reactive astrocytes, under pathological conditions (Martineau et al., 2013, 2014; Folorunso et al., 2023). The “serine shuttle model” outlines the key mechanisms governing D-Ser dynamics in the brain (Wolosker, 2011). The existence of a D-Ser shuttle from neurons to astrocytes within the hippocampus has been recently proposed (Abreu et al., 2023): D-Ser may accumulate in glial vesicles upon uptake from the extracellular medium, and is released in a SNARE-dependent manner to support the establishment of synaptic plasticity.

Astrocytes are the major source of L-Ser in the brain, which is *de novo* synthesized from the glycolytic intermediate D-3-phosphoglycerate through the phosphorylated pathway (PP) (Maugard et al., 2021). L-Ser is a main carbon source in the one-carbon metabolic pathway, and is required for the synthesis of proteins, nucleotides, phosphoglycerides, glycerides, sphingolipids, phosphatidylserine and methylenetetrahydrofolate, as well as of glycine (Gly) (Murtas et al., 2020). The PP is a metabolic sequence consisting of three enzymatic reactions catalyzed by: phosphoglycerate dehydrogenase (PHGDH), phosphoserine aminotransferase (PSAT) and phosphoserine phosphatase (PSP) (Murtas et al., 2020). By using differentiated astrocytes (see below), we recently reported that the enzymes of the PP produce a metabolic assembly (named “serinosome”), which likely undergoes clustering in response to varying metabolic stimuli (Rabattoni et al., 2023). We estimated that 47%–58% of the PP enzymes colocalize in the cytosol and that, based on size, each serinosome should contain a number of proteins in the 10^3^ to 10^5^ range with an average 2:2:1 PHGDH:PSAT:PSP stoichiometry. Thus, as it has been reported for PHGDH (catalyzing the rate-limiting step), altered levels of expression and activity of the PP enzymes are expected to perturb the equilibrium of the free vs. complexed form of the PP enzymes, likely contributing to distorted glial-neuron communication and, eventually, to NMDAR function impairment (Rabattoni et al., 2023). Actually, mutations in genes coding for enzymes of the PP are associated with several neurological disorders (Murtas et al., 2020), indicating the relevance of serine synthesis for brain functionality. The expression levels of the PP enzymes in the hippocampus have been recently investigated to shed light on brain metabolism during healthy aging vs. AD onset (Le Douce et al., 2020; Maffioli et al., 2022).

Relevant differences are known between human and mouse enzymes involved in D-amino acid metabolism. Various studies have established that mouse D-amino acid oxidase differs from its human counterpart (Molla et al., 2006; Koga et al., 2017), as well as mouse and human D-aspartate oxidase (Katane et al., 2017; Molla et al., 2020; Puggioni et al., 2020). However, little is known about the synthetic pathway in mice, as studies have primarily focused on human serine racemase (Raboni et al., 2018; Graham et al., 2019). Given these differences, as well as the broader distinctions between human and mouse biology (Li et al., 2021), we propose the generation of astrocytes from neural stem cells (NSCs) as a valid model to study L-Ser biosynthesis. We recently exploited the potential of a differentiation protocol based on the generation of human mature astrocytes from human fibroblasts reprogrammed to induced pluripotent stem cells (hiPSC) (Tripodi et al., 2023). The investigation of the proteomic and metabolomic profile during the differentiation process demonstrated that astrocytes at 30 days of differentiation are closer to mature rather than to reactive ones. We reported that axogenesis, pyrimidine metabolism, folate cycle and sphingolipid metabolism increased up to 30 days, as well as the intracellular levels of L-Ser, Gly, threonine, and L- and D-aspartate, while D-Ser synthesis was restricted.

Here, we used the same cellular model (hiPSC-derived astrocytes at 30 days of differentiation) to evaluate the impact on cell metabolism due to the overexpression of each of the three enzymes of the PP. The study was carried out on cells transduced with lentiviral particles to ectopically produce GFP-tagged proteins of the PP and employing a proteomic and metabolomic approach (also taking advantage of chiral HPLC for evaluating amino acid enantiomers). The results provide a deeper molecular understanding of the contribution of the PP enzymes to the astrocytic metabolism, specifically in relation to L-Ser metabolism.

## 2 Experimental procedures

### 2.1 Cell cultures

Human fibroblasts from ATCC [BJ (ATCC CRL-2522, Manassas, VA, USA)] were reprogrammed into hiPSC utilizing SENDAI virus carrying the embryonic genes OCT4, SOX2, KLF4 and cMYC, as previously reported (Tripodi et al., 2023). Pluripotency was verified by checking for the markers OCT4, SSEA4, SOX2 and NANOG. hiPSCs were then differentiated to Neural Stem Cells in Neural Expansion Medium composed of Neural Induction Medium and Advanced DMEM/F-12 Medium (Thermo Fisher) at 1:1 ratio, as described in Tripodi et al. (2023). NSCs were differentiated in astrocytes as in Tripodi et al. (2023) by seeding dissociated single cells at 25000 cells/cm^2^ density on Geltrex (Thermo Fisher 12760)-coated plates in astrocytes medium composed of DMEM 1X (Thermo Fisher 11995), 1% fetal bovine serum ES qualified (Thermo Fisher 16141), 2 mM Glutamax (Thermo Fisher 35050), 1% N2 supplement (Thermo Fisher 17502) and 1X antibiotic antimycotic solution (Thermo Fisher 15240). The medium was replaced with fresh medium every 3 to 4 days. When the cells reached 90%–95% confluency (approximately every 4-5 days), they were re-seeded to the initial density (25000 cells/cm^2^) as single cells in astrocyte medium, and cultured on Geltrex, following 5–10 min incubation with accutase (Thermo Fisher A1110501), pipetting and washing with DMEM.

Lentiviral vectors to obtain overexpression of GFP-tagged PHGDH, PSAT and PSP (together with the empty GFP vector) under a CMV promoter were purchased from Origene (Catalog numbers RC203949L4 for PHGDH, RC202475L4 for PSAT, RC209090L4 for PSP and PS100093 as control). For the packaging we used 3rd generation lentiviral vectors with improved biosafety. Lentiviral particles were produced in HEK-293T transfected with MDL, RSV, VSVG, and the plasmids for overexpression, by calcium phosphate transfection. Purification of lentivirus particles was performed by the PEG method. To quantify the lentiviral vector titer to calculate the MOI, HEK-293T cells were infected with serially diluted lentiviral samples and the expression of the GFP protein was measured using a fluorescent microscope. Each sample was analyzed in duplicates. Lentivirus transduction was performed in 27-days differentiated astrocytes. To increase the rate of virus-mediated transfer of nucleic acids into cells, astrocytes were transduced in suspension and then plated on geltrex-coated plates (MOI 1.5). In this condition the transduction rate after 72 h was approximately 65%. At 72 h after transduction (astrocytes at 30 days of differentiation), the cells were subjected to all further analysis described.

### 2.2 Western blot analysis

Human iPSC-derived astrocytes were homogenized in 60 μL of 0.2 M trichloroacetic acid, sonicated (three cycles, 10 s each) and then centrifuged at 13,000 *g* for 20 min. The supernatants were stored at −80°C for HPLC analysis. The pellets were solubilized in 1% SDS, sonicated (three cycles, 10 s each) and then centrifuged at 13,000 *g* for 20 min. The protein concentration in the supernatants was quantified using the Bradford reagent (500-0205, BioRad) and then these samples were analyzed by Western blot for the presence of PHGDH (rabbit anti-PHGDH, HPA024031, Sigma, dilution 1:1000), PSAT (rabbit anti-PSAT, abin2856767, Antibodies online, 1:1000), and PSP (rabbit anti-PSP, PA5-22003, Invitrogen, dilution 1:1000) (Maffioli et al., 2022). For each sample 20 μg of total proteins were separated by SDS-PAGE and then transferred on a PVDF membrane using the Mini Trans-Blot Cell system (BioRad). The membrane was blocked overnight at 4°C with 4% dried milk in Tris-saline buffer pH 8.0 added of 0.1% Tween 20 and subsequently incubated with primary antibodies diluted in 2% dried milk in Tris-saline buffer pH 8.0 added of 0.05% Tween 20 for 2 h at room temperature. After extensive washings, the membrane was incubated for 1 h at room temperature with anti-rabbit IgG (Alexa-Fluor Plus 800, 1:20000 dilution in Tris-saline buffer pH 8.0, 0.05% Tween 20). Membranes were analyzed by Li-cor ImageStudio software: the intensity signal of each sample was normalized by the GAPDH signal (detected using a mouse anti-GAPDH, 1:2000, MA5-15738 Invitrogen and a mouse IgG IRDye 680, 1:5000). Densitometric analysis was conducted on 5 biological replicates for each sample: control cells transduced with the empty GFP vector (CTR), and PHGDH^oe^, PSAT^oe^ and PSP^oe^ cells. The content of each protein was calculated by the software based on the intensity of known amounts of recombinant proteins and was related to the μg of total proteins loaded into the gel. Controls included the addition of a known amount of each recombinant protein to the samples (10 ng for PHGDH, and PSAT; 1 ng for PSP). Each sample was analyzed at least twice (in two different SDS-PAGE runs). The results were analyzed using Prism (Graphpad Software Inc.).

### 2.3 Confocal analysis

Immunofluorescence analysis was performed on transduced differentiated astrocytes (CTR, PHGDH^oe^, PSAT^oe^, and PSP^oe^; see above), seeded on Geltrex coated coverslips. The endogenous PHGDH, PSAT and PSP were stained using the rabbit anti-PHGDH (1:1000, HPA024031; Sigma); the mouse anti-PSAT (1:250, H00029968-A01; Abnova, Taipei, Taiwan), and the rabbit anti-PSP (1:250, PA5-22003; Invitrogen) primary antibodies, respectively, (diluted in PBS, 0.1% Triton X-100, 4% horse serum); and the Alexa Fluor 546 donkey-anti-mouse (A10036) and goat anti-rabbit (A11035) secondary antibodies (Invitrogen, 1:1000 in PBS, 0.1% Triton X-100). 30 days astrocytes fixed in PBS, 4% paraformaldehyde at room temperature for 10 min, were incubated in PBS, 0.2% Triton X-100, 4% horse serum, at room temperature for 20 min for block permeation, incubated at 4°C overnight in the primary antibodies and, after extensive washing, in the secondary antibodies at room temperature, for 1 h. Upon mounting using the Vectashield antifade mounting media (H-1700; Vector Laboratories), stained coverslips were imaged using an inverted laser scanning confocal microscope (TCS SP5; Leica Microsystems, Wetzlar, Germany) and a 63.091.25 NA plan apochromatic oil immersion objective. Image stacks (10 sections each, optimized thickness) were acquired in a sequential mode to avoid interference between the green (GFP) and the red (546) channels due to spectral overlap, without saturating any pixel. The signal/background ratio was adjusted by fixing the photomultiplier laser maximal levels of each channel, taking as reference controls prepared without adding primary antibodies.

Image analysis was performed using the open-source software FIJI.^1^ Acquired fields were processed by subtracting the background (default parameter settings) and applying the median filter. The colocalization and correlation of immunofluorescence signals were analyzed by the BIOP JACoP plugin. Regions of Interest (ROI) containing single cells were selected and processed by applying the Otsu threshold for both the red (endogenous PP’s enzymes) and the green (overexpressed GFP-tagged protein) channels. Overlapping (Mander’s 1 and 2, M1 and M2) and correlation (Pearson’s and Spearman’s) coefficients were determined. Manders’ colocalization coefficients measure the degree of co-occurrence independent of signal proportionality and provide an intuitive and direct metric of colocalization: M1 and M2 pair represent the fraction of the total pixels detected by channel 1 containing pixels detected by channel 2, and vice versa. On the other hand, Pearson’s correlation coefficient is a measure of the predictability of the linear relationship between the signal intensities in one image (channel 1) and the corresponding values in the other (channel 2), while the Spearman’s coefficient is a non-parametric measure of rank correlation and evaluates monotonic relationships, whether linear or not.

In order to assess whether the induced overexpression affects the serinosome formation, after fixation non-transduced (NEG), CTR, PHGDH^oe^, PSAT^oe^ and PSP^oe^ astrocytes were permeabilized with 0.2% Triton X-100 in PBS for 20 min at room temperature and subjected to the proximity ligation assay (PLA) using the Duolink In Situ Orange starter kit mouse/rabbit (DUO92008; Merck) as previously reported (Rabattoni et al., 2023). Briefly, coverslips were incubated in Duolink blocking solution for 60 min at 37°C and then overnight at 4°C with primary polyclonal antibody mixtures prepared in Duolink antibody diluent as follows: (a) rabbit anti-PHGDH (1:1000) and mouse anti-PSAT (1:250); (b) mouse anti-PSAT (1:250) and rabbit anti-PSP (1:250); (c) mouse anti-PHGDH (1:500, raised against the purified recombinant protein; Davids Biotechnologie, Regensburg, Germany) and rabbit anti-PSP (1:250). Coverslips were then incubated with PLA Probe MINUS and PLUS for 1 h at 37°C, with the ligation solution for 30 min at 37°C, and finally with the amplification solution for 100 min at 37°C. After each incubation, an extensive washing in Duolink buffer A was performed. Finally, cells were rinsed with Duolink buffer B and mounted with the provided DAPI-containing mounting medium to allow nuclei staining. Negative controls were prepared using a mixture of primary antibodies containing rabbit anti-PHGDH (1:1000) and mouse monoclonal anti-ERAB (1:500, sc-136326; Santa Cruz Biotechnology) antibodies. ERAB is a 27 kDa protein known to localize to the endoplasmic reticulum and the mitochondrial matrix (Nakayama et al., 2005), which therefore cannot interact with the cytosolic PHGDH. Images were acquired using Nikon A1R laser scanning confocal microscope with a 60× (NA 1.4) oil immersion objective. DAPI, GFP and PLA signals were detected by using a 405 nm, 488 nm and 543 nm laser light, respectively, for excitation, while the emitted signals were collected in the 425–475 nm, 500–550 nm and 570–620 nm range, respectively. The number of PLA spots/cell was determined by counting nuclei and PLA signals in selected fields containing 10–20 cells using the analysis particles function in FIJI after having properly adjusted the threshold of images and the parameter for particle detection.

### 2.4 Shotgun mass spectrometry analysis for label-free proteomics

The same astrocytes (CTR, PHGDH^oe^, PSAT^oe^, and PSP^oe^) were analyzed using a shotgun label free proteomic approach to identify and quantify protein expression. Cell lysis was performed in a urea-containing lysis buffer (20 mM HEPES pH 8.0, 8 M urea, and proteases and phosphatases inhibitor cocktail) (Coccetti et al., 2006, 2008). The homogenate was sonicated in 20–30 bursts using an ultrasonic probe and centrifuged at 16,060×*g* for 15 min at 18°C to remove cell debris. Prior to proteolysis, proteins were reduced with 13 mM dithiothreitol (15 min at 50°C) and alkylated with 26 mM iodoacetamide (30 min at room temperature). Samples were diluted to a final concentration of 1 M urea by addition of 20 mM HEPES, pH 8.0, and digested overnight with sequencing-grade trypsin (Promega) for 16 h at 37°C using a protein: enzyme ratio of 20:1 in the presence of 1 mM methylamine. The collected peptides were desalted using Zip-Tip C18 before Mass Spectrometric (MS) analysis as reported in Coccetti et al. (2006) 2008. NanoHPLC coupled to MS/MS analysis was performed on Dionex UltiMate 3000 directly connected to an Orbitrap Fusion Tribrid mass spectrometer (Thermo Fisher Scientific, Waltham, MA, USA) by a nanoelectrospray ion source. Peptide mixtures were enriched on 75 μm ID × 150 mm Acclaim PepMap RSLC C18 column and separated employing the LC gradient: 4% ACN in 0.1% formic acid for 3 min, 4%–28% ACN in 0.1% formic acid for 130 min, 28%–40% ACN in 0.1% formic acid for 20 min, 40%–95% ACN in 0.1% formic for 2 min and 95%–4% ACN in 0.1% formic acid for 3 min at a flow rate of 0.3 μL/min. MS spectra of eluting peptides were collected over an m/z range of 375–1,500 using a resolution setting of 120 000, operating in the data-dependent mode with a cycle time of 3 s between master scans. HCD MS/MS spectra were acquired in Orbitrap at resolution of 15,000 using a normalized collision energy of 35%, and an isolation window of 1.6 m/z. Dynamic exclusion was set to 60 s. Rejection of + 1, and unassigned charge states were enabled. The mass spectrometry proteomics data have been deposited to the ProteomeXchange Consortium via the PRIDE partner repository (Vizcaíno et al., 2016), with the dataset identifier PXD045073.

### 2.5 Enantiomeric HPLC

CTR (cells transduced with the empty GFP vector), PHGDH^oe^, PSAT^oe^, and PSP^oe^ astrocytes were homogenized in 60 μL of 0.2 M trichloroacetic acid (TCA), sonicated (three cycles, 10 s each) or in an ultrasound bath for 20 min and centrifuged at 13,000 *g* for 20 min at 4°C. The supernatant was used for HPLC analysis, and the pellets for Western blot analysis and protein quantification. For every single HPLC run (at least three for each sample), 5 or 10 μLof samples in TCA, were neutralized with NaOH and subjected to pre-column derivatization with o-phthaldialdehyde (OPA) and N-acetyl L-cysteine (NAC). The derivative diastereoisomers were separated on a Symmetry C8 reversed-phase column (5 μm, 4.6 × 250 mm or 3.5 μm, 4.6 × 150 mm, Waters, Milano, Italy) under isocratic conditions using 0.1 M sodium acetate buffer at pH 6.2 added with 1% tetrahydrofuran as mobile phase, at a flow rate from 0.7 to 1 mL/min (Punzo et al., 2016). Identification and quantification of all the amino acids analyzed were based on retention times and peak areas, related to those obtained with external standards. The identity of D-amino acids was confirmed by selective degradation after 4 h of incubation at 30°C with M213R RgDAAO variant (Sacchi et al., 2002). Amino acid content was normalized on total protein expressed in mg, determined by the Bradford assay as previously described (Western blot paragraph).

### 2.6 Database search and protein identification

Raw label-free MS/MS files from Thermo Xcalibur software (version 4.1) were analyzed using MaxQuant (version 1.6.0.1) and searched against the Human Uniprot sequence database (release 23/03/2023) (Tedeschi et al., 2017). Search parameters were as follows: initial maximum allowed mass deviation of 20 ppm for monoisotopic precursor ions and 0.5 Da for MS/MS peaks, trypsin enzyme specificity, a maximum of two missed cleavages, carbamidomethyl cysteine as fixed modification, N-terminal acetylation, methionine oxidation, asparagine/glutamine deamidation, and serine/threonine/tyrosine phosphorylation as variable modifications. False protein identification rate (1%) was estimated by searching MS/MS spectra against the corresponding reversed-sequence (decoy) database. The minimum required peptide length was set to 7 amino acids and the minimum number of unique peptides supporting protein identification was set to 1. Relative quantification was performed in MaxQuant’s built-in label-free quantification (LFQ) algorithm based on extracted ion intensity of precursor ions. Five biological replicates were carried out. Only proteins present and quantified in 80% of the repeats were considered as positively identified in a sample and used for statistical analyses performed by the Perseus software module (version 1.5.5.3).^2^ The relative expression level of PP’s enzymes was calculated using the built-in label-free quantification algorithms (LFQ) based on extracted ion intensity of precursor ions (Vernocchi et al., 2014). Variation of protein levels between different conditions was evaluated using the one-way ANOVA, Tukey’s HSD *post-hoc* test (*FDR ≤ 0.05, **FDR ≤ 0.01, ***FDR ≤ 0.001).

Focusing on specific comparisons (PHGDH^oe^ vs. CTR, PSAT^oe^ vs. CTR, PSP^oe^ vs. CTR and CTR vs. 30 days), proteins were considered differentially expressed if they were present only in one condition or showed significant *t*-test difference (Student’s *t*-test FDR ≤ 0.05). The comparison among CTR and astrocytes without GFP was carried out in the context of a parallel metadata analysis referring to the data of non-transduced astrocytes at 30 days of differentiation, previously published in Tripodi et al. (2023), that were acquired with the same instrument, in the same laboratory and under the same experimental conditions. Furthermore, the comparisons were made on the LFQ values internally normalized with the same software and the data were compared using a statistical tool (two samples *t*-test FDR ≤ 0.05) as recommended in the analyses of different data sets.

Bioinformatic analyses were carried out by Panther software (release 16.0) (Mi et al., 2013), String software (release 12.0) (Szklarczyk et al., 2023), and QIAGEN Ingenuity Pathway Analysis (IPA) (release July 2023), to cluster enriched annotation groups of Biological Processes, Molecular Function and Pathways within the set of identified proteins. Functional grouping was based on Fischer’s exact test *p*-value ≤ 0.05. Gene set enrichment analysis GSEA (release 4.3.2.) was applied to identify enriched Reactome pathways. The results were visualized by the Enrichment Map in Cytoskape (release 3.10.1) (*p*-value 0.005).

### 2.7 LC-MS metabolic profiling

Metabolomics analyses were performed essentially as in Tripodi et al. (2023), at the Sysbio Centre of Systems Biology, metabolomics facility in Milan, Italy. Cells were quickly rinsed with 0.9% NaCl and quenched with 500 μL ice-cold 70:30 acetonitrile-water. Plates were placed at −80°C for 10 min, then the cells were collected by scraping. Cells were centrifuged at 12,000 *g* for 10 min at 4°C. The supernatant was collected in a glass insert and dried as above. Samples were then resuspended with 150 μL of H_2_O before analyses. LC separation was performed using an Agilent 1290 Infinity UHPLC system and an InfinityLab Poroshell 120 PFP column (2.1 × 100 mm, 2.7 μm; Agilent Technologies). Mobile phase A was water with 0.1% formic acid. Mobile phase B was acetonitrile with 0.1% formic acid. The injection volume was 15 μL and LC gradient conditions were: 0 min: 100% A; 2 min: 100% A; 4 min: 99% A; 10 min: 98% A; 11 min: 70% A; 15 min: 70% A; 16 min: 100% A with 5 min of post-run. Flow rate was 0.2 mL/min, and the column temperature was 35°C. MS detection was performed using an Agilent 6550 iFunnel Q-TOF mass spectrometer with Dual JetStream source operating in negative ionization mode. MS parameters were: gas temperature: 285°C; gas flow: 14 L/min; nebulizer pressure: 45 psig; sheath gas temperature: 330°C; sheath gas flow: 12 l/min; VCap: 3700 V; Fragmentor: 175 V; Skimmer: 65 V; Octopole RF: 750 V. Active reference mass correction was through a second nebulizer using masses with m/z: 112.9855 and 1,033.9881 dissolved in the mobile phase 2-propanol-acetonitrile-water (70:20:10 v/v). Data were acquired from m/z 60–1,050. Data analysis and isotopic natural abundance correction were performed with MassHunter ProFinder (Agilent Technologies). Relative metabolite signals were determined after normalization to cell number and statistical analyses were performed using MetaboAnalyst 5.0 (Pang et al., 2021).

### 2.8 NAD^+^/NADH measurement

NAD^+^/NADH ratio was determined using the NAD^+^/NADH quantitation kit (Merck) according to the manufacturer’s instruction on astrocytes overexpressing each of the three PP enzymes.

## 3 Results

### 3.1 Human astrocytes overexpressing PHGDH, PSAT, and PSP

**FIGURE 1:**
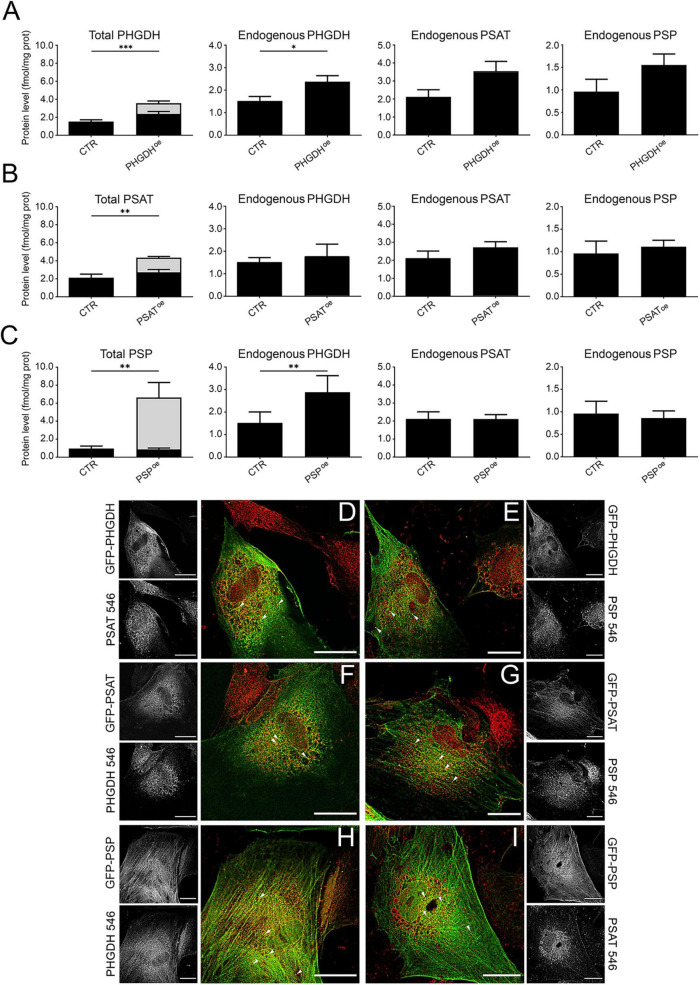
**(A–C)** PHGDH, PSAT and PSP protein levels in hiPSC-derived astrocytes determined by Western blot analysis. Comparison of the total and endogenous levels of the three enzymes after GFP-PHGDH **(A)**, GFP-PSAT **(B)** and GFP-PSP **(C)** overexpression. All panels: in black the endogenous level of the proteins are reported and in gray the amount of the GFP-tagged ones. CTR: control astrocyte, transduced with the empty GFP vector. Bars represent the mean ± SEM of values obtained from 5 sets of samples; *p < 0.05; **p < 0.01; ***p < 0.001. **(D–I)** Immunofluorescence and confocal analyses. The GFP signal (in green) associated with the overexpressed GFP-PHGDH **(D,E)**, GFP-PSAT **(F,G)**, and GFP-PSP **(H,I)**, is compared to the one corresponding to the endogenously expressed proteins stained with AlexaFluor546 (in red), as indicated in the different panels. In the grayscale panels the distribution of single-channel signals is shown. Scale bar = 25 μm. Arrowheads: examples of discrete spots of signal likely indicative of large structures formation.

Mature hiPSC-derived astrocytes (at 30 days of *in vitro* differentiation) (Tripodi et al., 2023) were transduced with lentiviral particles to overexpress GFP-tagged PHGDH, PSAT or PSP enzymes (namely PHGDH^oe^, PSAT^oe^ or PSP^oe^) as well as with GFP alone (as control, CTR). In order to discriminate the content of endogenous vs. overexpressed GFP-tagged PHGDH, PSAT, and PSP, the levels of the three enzymes were assessed by Western blot analysis based on their different mass (see Supplementary Figure 1). In control cells, 0.09 ± 0.03, 0.09 ± 0.04, and 0.024 ± 0.017 ng/μg total proteins for endogenous PHGDH, PSAT and PSP, respectively, were detected, and a total of 0.23 ± 0.04, 0.22 ± 0.05 and 0.32 ± 0.2 ng/μg total proteins for PHGDH, PSAT and PSP in PHGDH^oe^, PSAT^oe^ and PSP^oe^ cells, respectively. Notably, in all cases, the overexpression of each enzyme of the PP resulted in an increase of the levels of the three enzymes involved in the endogenous pathway (Figures 1A–C). Notably, the overexpression of PHGDH induced a statistically significant 1.5-fold increase of endogenous PHGDH level and a trend of increase for PSAT and PSP ones (not reaching a statistically significant threshold) (Figure 1A, gray bars). A statistically significant 2-fold increase of the endogenous PHGDH level was also observed in PSP^oe^ cells (Figure 1C).

In control astrocytes, as detected by immunofluorescence studies, endogenous PHGDH and PSAT were largely distributed in the cytoplasm with barely detectable levels in the nucleus, while the PSP signal was homogeneously distributed within the cells (Rabattoni et al., 2023). The overall distribution of PP enzymes was conserved when ectopically expressed as GFP-tagged proteins in PHGDH^oe^, PSAT^oe^ and PSP^oe^ astrocytes. The corresponding fluorescence was primarily diffused, although discrete signal spots, likely indicative of large structure formation, were also observed (Figures 1D–I, arrowheads). The size of the putative agglomerates was estimated based on their diameter (in the 0.5–0.7, 0.4–1.2, 0.5–1.4 μm range for GFP-PHGDH, GFP-PSAT and GFP-PSP, respectively): it belongs to the lower limit of the medium-size clusters (areas from 0.1 to 3 μm^2^) and is coherent with those recently reported in Rabattoni et al. (2023). In the same cells, the cellular localization of endogenous (untagged) PP enzymes was not altered: they showed a perinuclear distribution, with a low amount of the corresponding signals detected in the nucleus. No evidence for alternative subcellular localization was apparent, although their distribution appears to partially overlap with that of the cytoskeleton. This pattern is more pronounced in the case of overexpressed GFP-tagged proteins and will be explored in more detail in subsequent analyses. In the cytoplasm of transduced cells, the GFP signal associated with the ectopically expressed proteins largely overlapped with the ones corresponding to AlexaFluor546 staining for endogenous PHGDH, PSAT and PSP (Figures 1D–I): colocalization analyses indicated a consistent correlation between signals (see Pearson’s and Spearman’s coefficients in Table 1).

**TABLE 1:** Colocalization analysis.

Cells	Staining	Pearson’s	Spearman’s	M1	M2
PHGDH^oe^	PSAT 546	0.60 ± 0.13	0.74 ± 0.17	0.56 ± 0.12	0.59 ± 0.10
	PSP 546	0.48 ± 0.09	0.59 ± 0.11	0.56 ± 0.10	0.49 ± 0.11
PSAT^oe^	PHGDH 546	0.56 ± 0.07	0.061 ± 0.06	0.49 ± 0.15	0.61 ± 0.06
	PSP 546	0.37 ± 0.10	0.50 ± 0.13	0.49 ± 0.15	0.37 ± 0.07
PSP^oe^	PHGDH 546	0.61 ± 0.14	0.67 ± 0.14	0.64 ± 0.16	0.61 ± 0.10
	PSAT 546	0.49 ± 0.11	0.56 ± 0.13	0.56 ± 0.08	0.50 ± 0.13
No GFP	PP prot 546	0.14 ± 0.07	0.016 ± 0.08	0.05 ± 0.04	0.60 ± 0.12

The parameters related to signal co-occurrence (Manders’ overlap coefficients, M1 and M2), as well as to signals’ colocalization (Pearson’s and Spearman’s correlation coefficients), are listed. The reported values refer to the analysis of couples of signals corresponding to each GFP-tagged overexpressed PP’s protein and the endogenous PHGDH, PSAT and PSP, stained as in Figure 1D–I. “No GFP” represents transduced differentiated astrocytes in which the expression of the GFP-tagged PHGDH, PSAT or PSP is not detectable by immunofluorescence analyses.

Based on the protein levels determined by Western blot analysis (expressed as fmol/μg total proteins), a molar ratio of approximately 2:2:1 for PHGDH:PSAT:PSP was observed in non-transduced differentiated astrocytes. As indicated by Manders’ coefficients from immunofluorescence colocalization analysis, approximately 50% of the PP enzymes participate in forming the serinosome complex (Rabattoni et al., 2023). The overexpression of a single component of the PP altered the stoichiometry of the PHGDH:PSAT:PSP proteins in the cell, affecting the equilibrium between the free vs. serinosome complexed form. The observed PHGDH:PSAT:PSP molar ratios were 3.5:3.5:1.5, 1.8:4.0:1.0 and 3.0:2.0:6.5 for PHGDH^oe^, PSAT^oe^ and PSP^oe^, respectively (Figures 1A–C). Based on the signal correlation coefficients, GFP-PHGDH largely colocalized with endogenous PSAT (suggesting that 50%–60% of the two proteins was involved in a complex formation), and to a lower extent with endogenous PSP (Table 1): the lower signal colocalization for GFP-PHGDH and PSP was in line with PSP representing the minor component in the complex. This effect was also apparent in PSAT^oe^. Notably, when PSP was overexpressed, a pretty high colocalization of the corresponding signal with endogenous PHGDH ones was again observed (Table 1).

**FIGURE 2:**
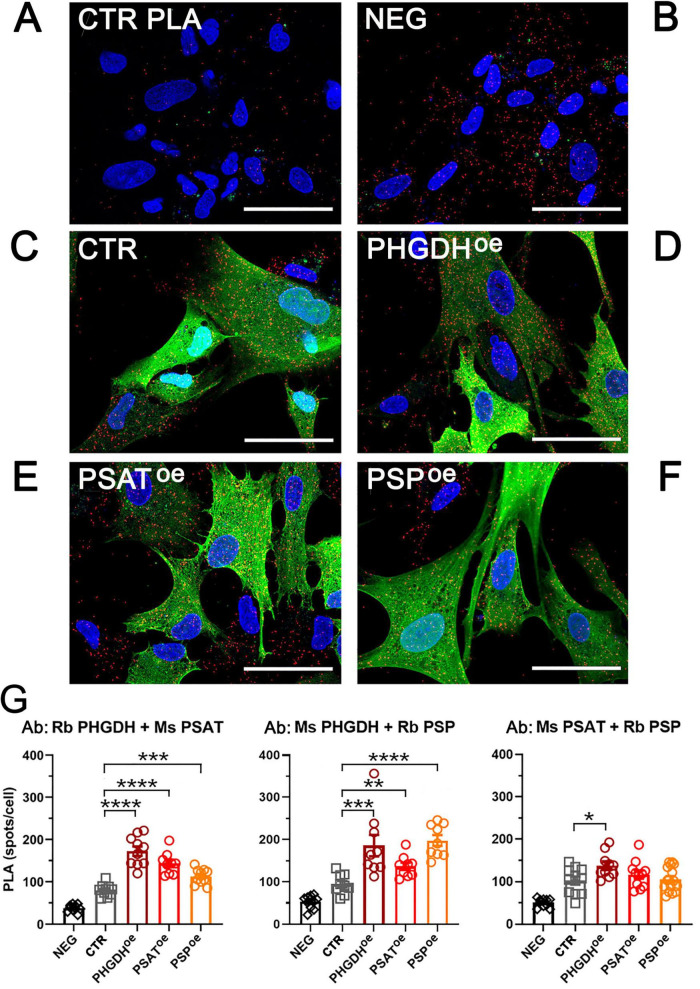
Detection of the serinosome formation by the PLA assay. Astrocytes were probed with the same antibody pairs used for immunofluorescence studies. **(A)** PLA negative control (CTR PLA): not transduced astrocytes were incubated with a primary antibody mixture containing rabbit anti-PHGDH and mouse anti-ERAB. **(B–F)** PLA signals detected in **(B)** non-transduced cells (NEG) and transduced astrocytes upon incubation with rabbit anti-PHGDH e mouse anti-PSAT antibodies: **(C)** control cells transduced with the empty GFP vector (CTR); **(D)** PHGDH^oe^, **(E)** PSAT^oe^ and **(F)** PSP^oe^ astrocytes. Red: proximity spots indicating the protein assembly formation. Green: GFP-tagged overexpressed PP’s proteins. Blue: nuclei stained with DAPI. Scale bar = 50 μm. The reported images are representative of sets of independent experiments. **(G)** Density of the PLA signal expressed as the number of spots determined by using the analysis particles function of the FIJI (IMAGEJ) software; *n* = 10 fields containing 10–20 cells each; error bars represent standard error of the mean (SEM). NEG, non-transduced astrocytes; CTR, control cells transduced with the empty GFP vector. *p < 0.05; **p < 0.01; ***p < 0.001; ****p < 0.0001.

Overall, confocal analyses indicate that the subcellular localization of GFP-tagged PP enzymes is unaltered compared to endogenous PHGDH, PSAT and PSP proteins and suggest that the expression of GFP-PHGDH (and to a limited extent of GFP-PSAT and -PSP) triggers additional serinosome formation. To further investigate this hypothesis, we performed PLA on PHGDH^oe^, PSAT^oe^ and PSP^oe^ cells. We previously used this assay to demonstrate the close proximity of the PP’s enzymes within the macromolecular clusters proposed to correspond to the serinosome (Rabattoni et al., 2023). Notably, all the cells overexpressing a component of the PP showed a statistically significant increase in the number of proximity spots per cell compared to control ones, transduced with the empty vector (CTR) (Figure 2), indicating that an increasing amount of protein molecules were engaged in protein complex formation. Under these conditions, the equilibrium of the proteins involved in the PP was shifted toward the serinosome-complexed one, also confirming a prominent role for PHGDH: actually, its overexpression accounts for the higher increase in PLA related signals (Figure 2G), pointing to a crucial role of this complicated enzyme in protein-protein recognition for serinosome generation.

### 3.2 Metabolomic and proteomic analyses of human astrocytes overexpressing PHGDH, PSAT and PSP

**FIGURE 3:**
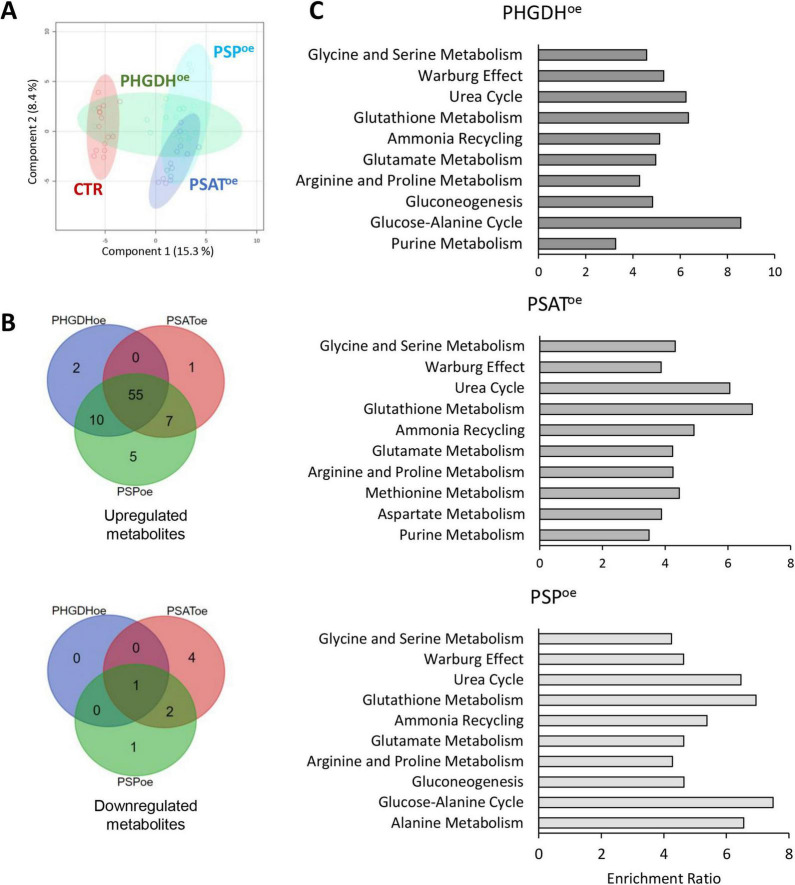
**(A)** PLS-DA and **(B)** Venn diagrams of astrocytes overexpressing PHGDH, PSAT and PSP. **(C)** Metabolite enrichment analysis of upregulated pathways in cells overexpressing PHGDH, PSAT or PSP compared to control cells. The enrichment was conducted using MetaboAnalyst 6.0 software.

The metabolomic and proteomic profiles of PHGDH^oe^, PSAT^oe^ and PSP^oe^ astrocytes were analyzed and integrated to evaluate the dynamic changes in endogenous metabolites and proteins, as well as the consequent perturbation of cellular pathways. A number of 89 differentially accumulated metabolites were identified by an untargeted metabolomics analysis by LC/MS (Supplementary Table 1). According to principal component analysis (PCA), there was a partial overlap among the three PHGDH^oe^, PSAT^oe^ and PSP^oe^ groups (Figure 3A). Most of the differential metabolites were upregulated with 55 of them common to all three groups (PHGDH^oe^, PSAT^oe^, PSP^oe^) and others increasing in at least two groups (Figure 3B), suggesting that the overexpression of any of the three enzymes of the PP leads to a common change of cellular metabolism. Only a small number of metabolites were found to decrease in cells overexpressing the PP enzymes, and interestingly they were mainly in PSAT^oe^ (Figure 3B), indicating that despite the common elicited response, peculiar features linked to the different enzymes are evident. In all the transduced differentiated astrocytes, the enrichment of metabolic pathways clearly showed that the most upregulated ones were mainly represented by amino acids (among which glycine and serine metabolism, as expected) and glutathione metabolism, along with urea cycle and Warburg effect (Figure 3C and Supplementary Table 1), confirming a general increase of metabolites closely associated to the PP.

**FIGURE 4:**
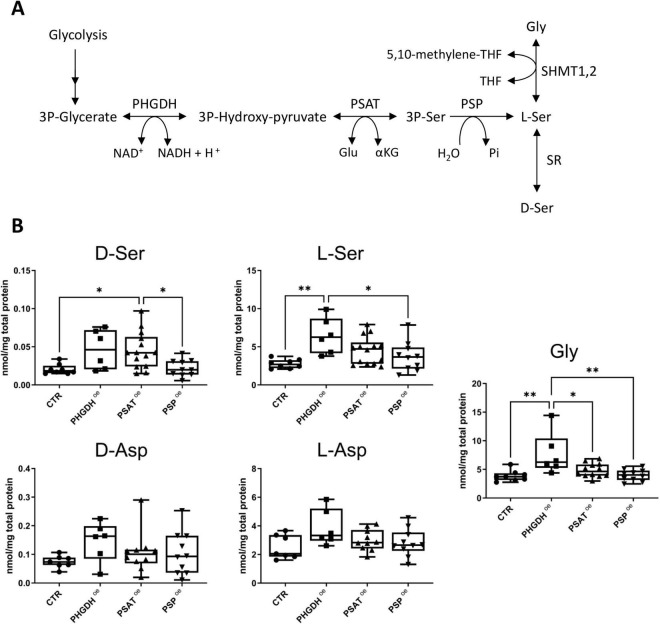
**(A)** Schematic representation of the phosphorylated pathway (PP). **(B)** Cellular concentration of D- and L-Ser, Gly, D- and L-Asp in control differentiated astrocytes transduced with the empty GFP vector (CTR) and in the ones overexpressing PHGDH, PSAT or PSP. The box plots show the median (central line), interquartile range (box) and minimum and maximum values (whiskers). Individual data points represent measurements from at least six biological replicates, each analyzed in three technical replicates. Statistical analysis performed: one-way ANOVA multiple comparison (Graphpad Prism 9.0) *p < 0.03, **p < 0.003.

To quantify the effect of PHGDH, PSAT or PSP overexpression on the levels of Ser and aspartate stereoisomers, as well as Gly, enantiomeric HPLC analysis was performed. In PHGDH^oe^ astrocytes a statistically significant increase in L-Ser and Gly concentrations was observed (2.3 and 2.0-fold increase, respectively, Figure 4B). In contrast, PSAT^oe^ astrocytes exhibited a less pronounced trend of increase in L-Ser and Gly levels compared to PHGDH^oe^ astrocytes (1.5- and 1.3-fold, respectively) and, compared to control, a statistically significant increase in D-Ser level. In PSP^oe^ astrocytes, a minor effect on Ser levels was detected, while Gly concentration was unaffected (Figure 4B). The changes in L-Ser (and Gly) levels did not reach statistical significance in either PSAT^oe^ or PSP^oe^ astrocytes, confirming the primary kinetic role of PHGDH in the PP (Rabattoni et al., 2023). Regarding aspartate levels, an increase trend (never reaching a statistical significance) was apparent for both enantiomers in PHGDH^oe^ astrocytes. Interestingly, our analyses confirm that D-Asp levels are higher than D-Ser ones in hiPSC-derived astrocytes, as previously reported (Tripodi et al., 2023).

**FIGURE 5:**
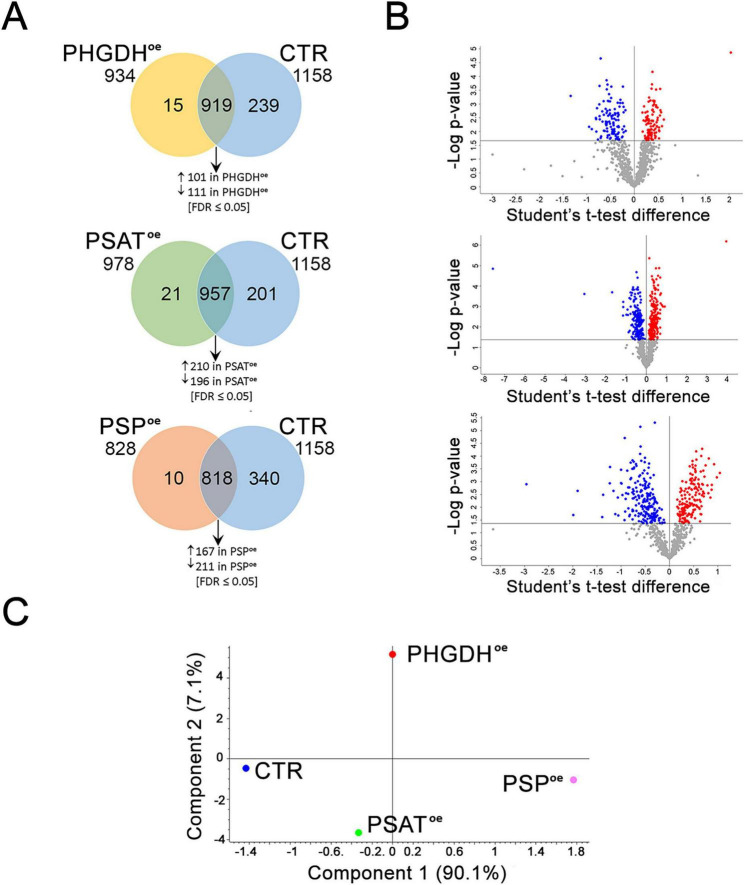
Proteomic analysis of PHGDH^oe^, PSAT^oe^ and PSP^oe^ astrocytes. **(A)** Venn diagrams of PHGDH^oe^ vs. CTR, PSAT^oe^ vs. CTR, PSP^oe^ vs. CTR and **(B)** Volcano plots of the differentially abundant proteins identified using Student’s *t*-test (FDR ≤ 0.05). Each protein is represented as a dot and is mapped according to its fold change on the ordinate axis, with the *p*-value by *t*-test on the abscissa. The red and blue dots indicate increased and decreased proteins in each comparison. Gray dots do not meet the FDR criteria. **(C)** PCA analysis of the PHGDH^oe^, CTR, PSAT^oe^ and PSP^oe^ proteomes.

The proteomes of PHGDH^oe^, PSAT^oe^ or PSP^oe^ astrocytes were characterized by a quantitative shotgun label-free strategy. The Venn diagrams and the Volcano plots of the analysis are reported in Figures 5A, B, respectively. A principal component analysis (PCA) on the 4 data sets (CTR, PHGDH^oe^, PSAT^oe^ and PSP^oe^) from 5 biological replicates clearly showed that the overexpression of each enzyme significantly alters the astrocytes proteome in a different and peculiar way (Figure 5C).

The analysis carried out to identify major differences versus astrocytes overexpressing GFP as a control, showed proteins exclusively expressed in each condition, as well as proteins statistically different, and proteins which did not change their expression level in the various comparisons (PHGDH^oe^ vs. CTR, PSAT^oe^ vs. CTR, PSP^oe^ vs. CTR) (Supplementary Tables 2–4 and Figures 5A, B). Focusing on these comparisons, the proteins differentially or exclusively expressed were analyzed by Panther (Supplementary Table 5) to find enrichment in biological processes (GOBP) and pathways. The data sets were further analyzed by QIAGEN Ingenuity Pathway Analysis (QIAGEN IPA, Supplementary Table 6) for a deeper interpretation. Proteomic analysis of PP enzymes highlighted significant differences in PHGDH^oe^, PSAT^oe^ or PSP^oe^ astrocytes. The expression of serine hydroxymethyltransferase 2 (SHMT2), which connects Ser to Gly (Figure 4A), did not show any significant difference in the three conditions. For all comparisons, the enzymes involved in the D-Ser metabolism, i.e., SR and D-amino acid oxidase (DAAO), and the D-Asp degrading enzyme D-aspartate oxidase (DASPO) were not detected by LC–MS/MS, suggesting that their expression level in differentiated astrocytes was below the detection limit of the instrumental setup.

The proteome of CTR astrocytes overexpressing GFP and of the non-transduced ones at 30 days of differentiation, previously published in Tripodi et al. (2023), were compared to evaluate the impact of the GFP heterologous expression (and/or of the lentiviral transduction process) and rule out whether the proteome alterations observed in PHGDH^oe^, PSAT^oe^ and PSP^oe^ astrocytes may be ascribed to the presence of GFP (Supplementary Table 7). The analysis by Perseus identified proteins differentially expressed in the two datasets: 399 and 1,667 proteins were increased or exclusively expressed in CTR and non-transduced astrocytes at 30 days of differentiation, respectively (Supplementary Table 7). Gene Set Enrichment Analysis (GSEA) performed on these proteins identified over-represented protein classes in terms of biological processes (GOBP) and pathways (REACTOME). As shown in Supplementary Figure 2, which represents the enrichment map of the REACTOME GSEA analysis, GFP overexpression mainly increased proteins involved in collagen and extracellular matrix organization while decreased those participating in actin folding, and morphogenesis. Notably, proteins related to metabolism and the PP pathway remained unaffected.

### 3.3 Focus on glycolysis and TCA

**FIGURE 6:**
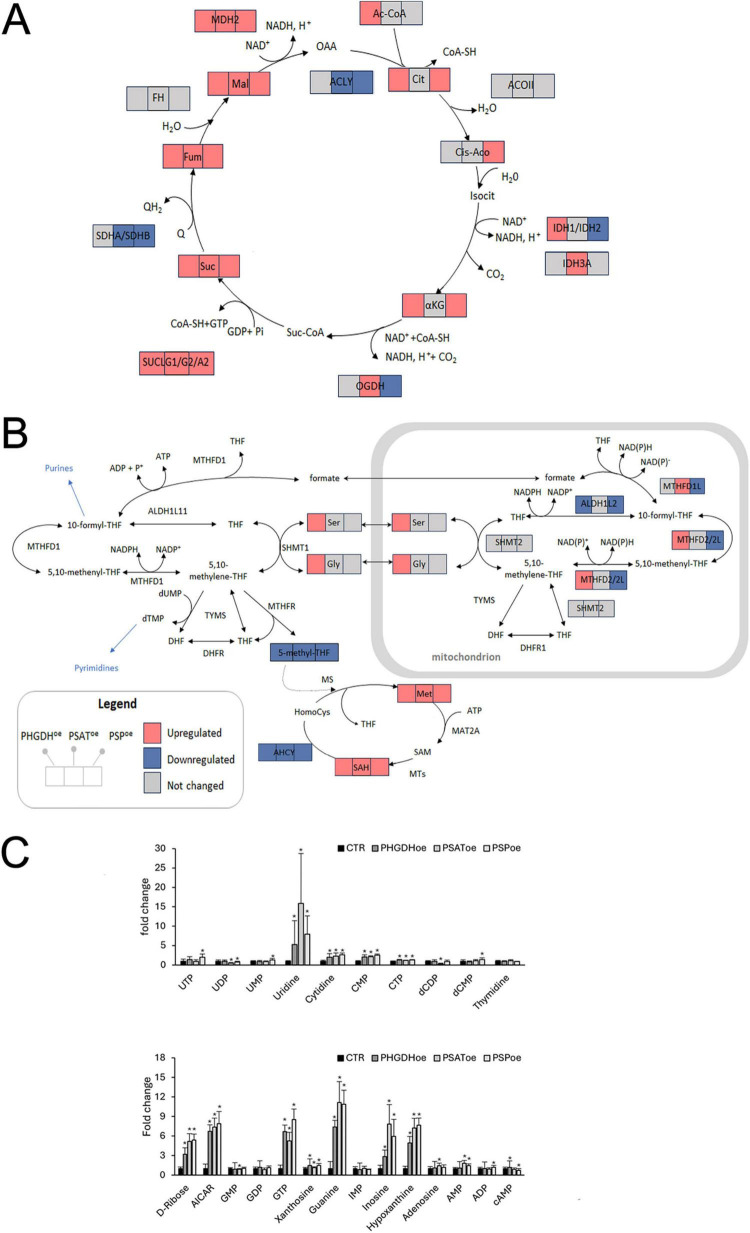
**(A,B)** Schematic representation of **(A)** the TCA cycle and **(B)** the one-carbon metabolism; squares show detected metabolites/enzymes and colors indicate their significant (*p* > 0.05) variations upon overexpression of the PP proteins. The proteins are indicated by the official gene code. TCA cycle: ACLY, ATP-citrate synthase; ACOII, aconitate hydratase; IDH1, isocitrate dehydrogenase (NADP) cytoplasmic; IDH2, isocitrate dehydrogenase (NADP), mitochondrial; IDH3A, isocitrate dehydrogenase (NAD) subunit alpha; OGDH, 2-oxoglutarate dehydrogenase; SUCLG1, succinate-CoA ligase (ADP-forming) subunit alpha; SUCLG2, succinate-CoA ligase (GDP-forming) subunit beta; SUCLA2, succinate-CoA ligase (ADP-forming) subunit beta; SDHA, succinate dehydrogenase (ubiquinone) flavoprotein subunit; SDHB, succinate dehydrogenase (ubiquinone) iron-sulfur subunit; FH, fumarate hydratase; MDH2, malate dehydrogenase; one carbon metabolism: MTHFD1/1L, methylenetetrahydrofolate dehydrogenase 1/1L mitochondrial; MTHFD2/2L, methylenetetrahydrofolate dehydrogenase 2/2 L mitochondrial; ALDH1L1, aldehyde dehydrogenase 1 family member L1 cytosolic; ALDH1L2, aldehyde dehydrogenase 1 family member L2 mitochondrial; SHMT1, serine hydroxymethyltransferase 1 cytosolic; SHMT2, serine hydroxymethyltransferase 2 mitochondrial; TYMS, thymidylate synthase; DHFR2, dihydrofolate reductase 2, mitochondrial, MAT2A, S-adenosylmethionine synthase isoform type-2; MTs, methyltransferases; AHCY, adenosylhomocysteinase, DHFR, dihydrofolate reductase; MTHFR, methylenetetrahydrofolate reductase (NADPH); TYMS, thymidylate synthase. **(C)** Levels of metabolites related to pyrimidine and purine metabolism. Data are presented as mean ± standard deviation, *p < 0.05.

L-Ser biosynthesis starts from D-3-phosphoglycerate, and α-ketoglutarate, produced with the PSAT catalyzed reaction, can fuel the TCA cycle (Figure 4A). Accordingly, we analyzed changes in the metabolic pathways of glycolysis and tricarboxylic acid (TCA) cycle due to PP enzymes overexpression. The cells overexpressing any of the three PP enzymes showed higher levels of glucose, glucose-6-phosphate (Glu6P), fructose-1,6-bisphosphate (Fru1,6bP) and pyruvate (with the exception of Fru1,6bP and pyruvate in PSAT-overexpressing cells), as well as pyruvate-deriving metabolites, such as lactate and alanine (Supplementary Table 1). Furthermore, metabolites of the TCA cycle from succinate to oxaloacetate increased in all samples (Figure 6A). In PHGDH^oe^ astrocytes, the proteomic analysis by Panther showed a significant increase in β-oxidation of fatty acids and TCA cycle, promoting the ATP synthesis coupled to the electron transport chain (Supplementary Table 5), in keeping with the upregulation of TCA cycle metabolites, as shown in Figure 6A. IPA analysis confirmed the increase of oxidative phosphorylation (Supplementary Table 6) and highlighted the enhancement of glucose uptake (higher GLUT1 expression, in accordance with the higher intracellular level of glucose, Glu6P, and Fru1,6bP, shown in Supplementary Table 1) and NAD signaling pathway. Moreover, proteins involved in the mitochondrial respiration such as apoptosis-inducing factor mitochondrial-associated (AIFM1), COX5A, ETFB, PHB2, increased in PHGDH^oe^ astrocytes. Among these, AIFM1 is particularly noteworthy. This NADH dehydrogenase, located in the mitochondrial intermembrane space, operates alongside the respiratory chain to reoxidize NADH without contributing to proton pumping. Its valve-like function within the respiratory chain plays a crucial role in preventing oxidative damage under physiological conditions and acts as a sensor of mitochondrial metabolic fluxes (Supplementary Table 2 and Supplementary Figure 3) (Wischhof et al., 2022).

Another key protein involved in mitochondrial pyruvate metabolism, energy production and redox balance is the mitochondrial malic enzyme ME2, whose level decreased in PHGDH^oe^ astrocytes (Supplementary Table 2 and Supplementary Figure 3). This enzyme, which is dependent on either NAD^+^ or NADP^+^, catalyzes the conversion of L-malate to pyruvate, concurrently reducing NAD(P)^+^ to NAD(P)H and lowering the cellular NAD^+^/NADH ratio. ME2 functions as an energy sensor and plays a role in glucose and insulin secretion, as well as being essential for glutaminolysis in rapidly proliferating tissues. Under physiological conditions, ME2 activity is high in synaptic mitochondria in the brain, suggesting its involvement in the pyruvate recycling pathway and in maintaining reduced glutathione levels in synaptic terminals. Despite disruptions in NAD^+^/NADH-related processes, the levels and intracellular ratio of these cofactors remained unchanged, indicating the existence of compensatory mechanisms that preserve NAD^+^/NADH homeostasis (Supplementary Figure 4).

Most of the pathways and biological processes increased in PHGDH^oe^ astrocytes were also enriched in PSAT^oe^ ones, such as TCA cycle and aerobic respiration, as shown in Supplementary Tables 5, 6. The overexpression of PSP resulted in a proteome modulation, straddling the condition induced by PHGDH and PSAT ectopic expression. Also in this case, mitochondrial proteins were among the most modulated ones by overexpression. In the α-ketoglutarate/Glu exchange fluxes, glutamate dehydrogenase (GLUD1), ornithine aminotransferase (OAT) and aspartate aminotransferase (GOT2) increased in line with the risen levels of metabolites of TCA and urea cycles (Supplementary Table 1 and Figures 3C, 6A). In contrast to PHGDH^oe^, a decrease in the levels of enzymes involved in glycolytic processes was observed in both PSAT^oe^ and PSP^oe^ astrocytes (Supplementary Tables 3–6). Consistently, only PSAT^oe^ and PSP^oe^ showed an increase in mitochondrial aldehyde dehydrogenase (ALDH1B1), an enzyme involved in acetaldehyde metabolism and the maintenance of glucose homeostasis (Singh et al., 2015). Similar to PSAT^oe^, PSP^oe^ also triggered an upregulation of proteins involved in cristae formation and inner mitochondrial membrane organization, consistent with the marked increase in the mitochondrial respiration activity linked to the TCA cycle activation.

### 3.4 Focus on amino acids metabolism

As reported in Supplementary Table 6, PHGDH overexpression led to the enhanced expression of five proteins of the GABAergic receptor signaling pathway. Among these, two are subunits of the vacuolar cAMP-dependent protein kinase (PRKAR1A and PRKAR2A), which in astrocytes represents one of the two major transducer of cAMP signals -alongside EPAC1- and increases glucose uptake and glycogenolysis thereby triggering glycolysis and energy production. The other three proteins were aspartate aminotransferase (GOT2), glutaminase (GLS) and glutamate dehydrogenase GLUD1 [NAD(P) dependent], all of which regulate the levels of the neurotransmitter Glu. Consistently, Asp, Glu and α-ketoglutarate levels increased (Supplementary Table 2 and Figure 3C). Moreover, GOT2 facilitates the cellular uptake of long-chain free fatty acids, which can fuel the fatty acid β-oxidation in PHGDH^oe^ astrocytes. GOT2, along with GLS and GLUD1 also contribute to the biosynthesis of Arg, whose transporter, CAT1, was significantly increased in PHGDH overexpressing cells (Supplementary Table 3), in parallel with an increase in intracellular Arg level (Supplementary Table 1). CAT1 was the only amino acid transporter found to be upregulated in PHGDH^oe^ astrocytes, whereas SLC1A5 (neutral amino acid transporter, ASCT2) was the only one downregulated (Supplementary Table 3). CAT1, is a rate-limiting regulator of the mTOR pathway that controls neuronal growth by regulating the cytoskeleton dynamics and neuronal process extension. Accordingly, our findings suggest that PHGDH overexpression leads to reduced activity of the mTOR pathway, impaired cytoskeleton remodeling and decreased protein biosynthesis, while concurrently an increase of CAT1 expression was observed (Supplementary Table 6). Interestingly, although the preferred substrate of the alanine, serine, cysteine transporter 2 (ASCT2) is the amino acid glutamine (Gln), it has been proposed that this transporter mediates glutamate (Glu) reuptake in astrocytes in exchange for Gln. Once inside neurons, Gln is then used to synthesize Glu and GABA, both of which function as neurotransmitters. ASCT2 was long considered responsible for D-Ser distribution in the brain, until Wolosker’s group demonstrated that ASCT1 is, in fact, the primary physiological transporter for this D-amino acid (Kaplan et al., 2018). Moreover, ASCT2 expression is associated with mTOR pathway activation and was found to be reduced in our PHGDH^oe^ dataset (Supplementary Table 2).

PSAT overexpression led to increased expression of the arginine transporter CAT1, the glucose transporter GLUT1, and the nitrogen cycle enzymes GLUD1 and OAT (Supplementary Table 3 and Supplementary Figure 3), consistent with the findings of Hwang et al. (2016), suggesting that PSAT plays a role in regulating intracellular α-ketoglutarate levels.

In PSP^oe^ cells, the α-ketoglutarate/glutamate exchange fluxes showed increased levels of GLUD1, OAT, and the aspartate aminotransferase GOT2, consistent with the metabolomic data (Supplementary Figure 3 and Supplementary Table 1) (Alberghina and Gaglio, 2014; Hwang et al., 2016).

### 3.5 Focus on one-carbon metabolism

Other key metabolites closely linked to the serine pathway include those involved in one-carbon and glutathione metabolisms. Most of these metabolites were upregulated following overexpression of PHGDH, PSAT, and PSP, whereas 5-methyltetrahydrofolate—a component of the folate cycle—was the only metabolite strongly downregulated in all transduced differentiated astrocytes (Supplementary Table 1 and Figure 6B). Folate-mediated one-carbon metabolism (FOCM) occurs in both the cytoplasm and mitochondria, with strict compartmentalization ensured by distinct isoforms of the enzymes specific to each compartment (Sun et al., 2023) (Figure 6B). In this process, a one-carbon unit from Ser is transferred to tetrahydrofolate (THF) to form 5,10-methylene-THF, which is then oxidized to 5,10-methenyl-THF by methylenetetrahydrofolate dehydrogenase 1/1L (MTHFD1 is the cytosolic isoform and MTHFD1L is the mitochondrial one) and methylenetetrahydrofolate dehydrogenase 2/2L (MTHFD2/2L, both localized in mitochondria). 5,10-Methylene-THF can also be reduced to 5-methyl-THF by MTHFR in the cytosol. 5-Methyl-THF is subsequently demethylated to regenerate tetrahydrofolate, completing the cycle (Figure 6B). PHGDH overexpression selectively modulated the expression of mitochondrial isoforms of two key enzymes: it upregulated MTHFD2/2L and downregulated aldehyde dehydrogenase 1 family member L2 (ALDH1L2), a strictly NADP^+^-dependent enzyme and a major source of mitochondrial NADPH (Supplementary Table 2). The formate produced via this pathway can be exported to the cytosol, converted into 10-formyl-THF, and directed into the purine biosynthesis pathway—consistent with metabolomic data showing upregulation of purine metabolism intermediates (Figure 6C).

The scenario was markedly different in PSAT^oe^ astrocytes. Levels of MTHFD2/2L and ALDH1L2 remained unchanged, whereas the mitochondrial aldehyde dehydrogenase ALDH1B1—an enzyme involved in acetaldehyde metabolism and glucose homeostasis—was upregulated. In addition, MTHFD1L was significantly increased. This enzyme oxidizes 10-formyl-THF in the mitochondria, generating both NADPH and formate; the latter can be exported to the cytosol to support the purine and methionine cycles (Supplementary Table 3). Consistently, metabolomic data revealed elevated levels of cysteine (Cys), S-adenosylhomocysteine (SAH), and AMP (Figures 6B, C and Supplementary Table 1).

The most notable differences among PHGDH^oe^, PSAT^oe^ and PSP^oe^ astrocytes were as follows: PHGDH overexpression led to increased MTHFD2/2L and decreased levels of ALDH1L2, whereas both enzyme remained unchanged in PSAT^oe^ and were downregulated in PSP^oe^ (Figure 6B and Supplementary Tables 3, 4). In contrast, MTHFD1LL—upregulated in PSAT-overexpressing astrocytes— was unchanged in PHGDH^oe^ and downregulated in PSP^oe^.

5-methyl-THF is directly utilized for methionine (Met) synthesis by the enzyme methionine synthase (MS; Figure 6B). Although MS and methionine adenosyltransferase 2A (MAT2A) were below the detection threshold in our analysis, the marked reduction in 5-methyl-THF levels, alongside the observed increase in Met and S-adenosylhomocysteine (SAH) (Figure 6B), strongly suggests that the overexpression of each of the three PP enzymes affects methylation processes in astrocytes. Supporting this, the significant downregulation of adenosylhomocysteinase (AHCY)—the sole enzyme in mammals responsible for the reversible conversion of SAH to adenosine and L-homocysteine (Vizán et al., 2021)—suggests a potential alteration in the S-adenosylmethionine (SAM):SAH ratio. Such an imbalance may impair the transmethylation capacity for specific substrates and contribute to replication stress (Vizán et al., 2021).

Given the essential role of folates in nucleotide biosynthesis, we also examined purine and pyrimidine metabolism. In all three experimental conditions, several metabolites from both pathways were predominantly upregulated (Figure 6C). Although the expression of 5′-nucleotidase (NT5E) remained unchanged across comparisons, the overexpression of PP enzymes primarily led to a significant increase of purine bases (inosine, hypoxanthine, and guanine) and the pyrimidine base uridine. The levels of nucleotides were generally less pronounced than their corresponding nucleobases, with the notable exception of GTP, which exhibited a marked signal (Figure 6C).

## 4 Discussion

In past years, a number of studies focused on the investigation of the effects due to PHGDH inhibition, especially on the metabolism of cancer cells related to decreased L-Ser levels (Pacold et al., 2016; Gao et al., 2018; Reid et al., 2018). In this paper, for the first time, we present data on an efficient overexpression of each of the three enzymes of the PP in hiPSC–derived differentiated human astrocytes, which are crucial for fueling L-Ser to neurons in the brain. We recently demonstrated that in this cell type the three enzymes can cluster in a transient and highly dynamic metabolic assembly, the putative “serinosome,” that provides a metabolite channeling solution for the control of L–Ser biosynthesis (Rabattoni et al., 2023).

Here we show that when each enzyme of the PP is individually ectopically expressed, it is largely present in the cytoplasm and partially organized in the serinosome (Figures 1, 2 and Table 1). Notably, PHGDH overexpression is providing the highest increase in co-localization and stabilization of the complex with endogenous PSAT and PSP proteins (Figure 2): this agrees with the elaborated structure of the dehydrogenase. PHGDH is a homotetrameric protein and is made by four domains, two of them (the aspartate kinase-chorismate mutase-tyrA prephenate dehydrogenase, ACT, and the allosteric substrate-binding, ASB) are relevant for modulation of the oligomeric state and protein-protein recognition (Riva et al., 2024). Even PSP overexpression (the minor component of the serinosome, endogenously expressed at low levels) results in endogenous PHGDH accumulation, further pointing to an interaction between these two components of the serinosome. Based on increased L-Ser levels (Figure 4B), our data suggest that the alteration in stoichiometry of the PP enzyme levels due to the ectopic overexpression does not seem to affect the serinosome functionality.

The ectopic expression of each enzyme of the PP increases the L-Ser cellular concentration, reaching a statistically significant threshold for PHGDH only (Figure 4B). This is in agreement with the kinetic studies on the *in vitro* reconstructed PP (Rabattoni et al., 2023), showing that PHGDH is rate-limiting in L-Ser synthesis, as well as PSAT (under selected conditions of substrates and cofactors concentrations), while PSP has a minor kinetic effect on L-Ser production (mainly acting on the overall equilibrium). On this side, the strict connection between Ser and Gly metabolism is evident (Figure 4).

A trend toward an increase in D-Ser levels is observed in both PHGDH^oe^ and PSAT^oe^, with statistical significance reached only in the latter (Figure 4B). However, quantification is challenging due to low concentrations. Additionally, SR levels are below the detection limit of our analyses, which aligns with existing literature indicating that SR is primarily present in neurons and reactive astrocytes, rather than in mature astrocytes (Wolosker, 2011; Folorunso et al., 2023).

Comprehensive reviews of the metabolism of astrocytes have been recently published (Maugard et al., 2021; Andersen et al., 2022) and these works represent relevant references for our results. A consistent increase of glycolytic and pyruvate-deriving metabolites, as well as TCA cycle and mitochondrial activities, is a common picture when PP enzymes are overexpressed. A recent report indicates that short-term activation of astroglial type-1 cannabinoid (CB1) receptors induces a metabolic shift in glycolysis, promoting lactate-dependent D-Ser production (Fernández-Moncada et al., 2024). Furthermore, activation of the lactate receptor HCAR1 by lactate has been shown to stimulate glycolysis, generating lactate from pyruvate and L-Ser from 3PG. In this way, lactate may control synaptic D-Ser, modulating signaling through NMDARs. Lactate could regulate synaptic D-Ser availability also modulating the PP through an unknown mechanism. Nevertheless, the integration of the metabolomic and proteomic data highlights some peculiarities among the three conditions mainly related to the one-carbon metabolism in mitochondria (Figure 6B). The intracellular level of 5-methyl-THF is always downregulated and although the analysis does not discriminate between the cytosolic pool and the mitochondrial one, only the mitochondrial isoforms of the enzymes involved in its metabolism are differently regulated. Indeed, MTHFD2/2L level is higher in PDGDH^oe^ and lower in PSP^oe^ astrocytes. On the contrary MTHFD2/2L is not modulated in PSAT^oe^ and this is also the case of mitochondrial aldehyde dehydrogenase ALDH1L2, which instead is downregulated in both PHGDH^oe^ and PSP^oe^ cells. The only two enzymes upregulated in PSAT^oe^ are MTHFD1L, which level is lower in PSP^oe^ and unchanged in PHGDH^oe^, and the mitochondrial aldehyde dehydrogenase ALDH1B1, which level increases in PSAT^oe^ and in PSP^oe^.

The serine-glycine-one carbon metabolism (SGOC) pathway is interconnected with the Met cycle through MTHFR, which catalyzes the irreversible conversion of 5–10-methylene-THF to 5-methyl-THF, the only metabolite significantly decreased in astrocytes ectopically expressing PHGDH, PSAT and PSP (Supplementary Table 1 and Figure 6B). As shown in Figure 6B, in PHGDH^oe^ the significant opposite effect on MTHFD2/2L and ALDH1L2, the only two enzymes highly modulated in that condition, may be framed in the general control of redox homeostasis, in agreement with recent studies reporting that both cytosolic and mitochondrial methylenetetrahydrofolate dehydrogenases contribute to regulate the redox state of cells (Sun et al., 2023). On the contrary, in PSAT^oe^ the significant increase of MTHFD1L, which in mitochondria hydrolyzes 10-formyl-THF to formate that can be thus conveyed to the cytoplasm, suggests that the one carbon metabolism fuels mainly the anabolic processes, such as amino acids and protein synthesis, following the behavior usually described for the folate metabolism.

In mouse models of AD an increase in PHGDH mRNA level has been reported in hippocampal astrocytes (Chen et al., 2022). In the same study, a sequential increase of PHGDH expression in patients with early and late AD pathology was reported in hippocampus and prefrontal cortex: the increase in PHGDH expression correlated with progression of clinical symptoms and worsening cognitive decline. Although different mechanisms in early and late stages of AD have been reported, highlighting a main role of a reduced glycolytic flux at the onset of the disease (Le Douce et al., 2020), a similar increase in PHGDH level has been also observed in the hippocampus of AD patients, especially in women, as a mechanism to counteract the decrease in Ser enantiomers level observed under AD conditions (Maffioli et al., 2022). AD has been linked to D-Ser through two apparent physiopathological processes: (a) an excitotoxic scenario as Aβ oligomers promote SR expression (Kundu and Dubey, 2021); (b) a failure in brain aerobic glycolysis resulting in an impaired L-Ser and D-Ser production, leading to abnormal synaptic plasticity and cognitive performance (Le Douce et al., 2020). According to the latter hypothesis, in the early phase of the disease the NMDA receptor-mediated signaling can benefit from increased D-Ser/Gly co-agonist levels. Here, we confirm that an increase of PHGDH expression determines an evident impact on L-Ser metabolism in astrocytes and that a strong metabolic reprogramming is a common feature when each of the three enzymes of the PP is overexpressed in hiPSC-derived astrocytes.

The increased PHGDH expression in AD models and patients brain tissues (e.g., 1.5–2.5-fold increase in AD mouse models and 1.54-fold in the hippocampus of AD patients) (Chen et al., 2022; Maffioli et al., 2022), supports the notion that the PP is modulated in pathological conditions. However, whether this upregulation serves as a driver of astrocyte reactivity or represents part of a broader adaptive response remains to be clarified. In our model, the overexpression of PP enzymes primarily alters mitochondrial proteins without inducing classical markers of astrocyte reactivity—such as those listed in Tripodi et al. (2023)—nor the markers described in TIC-induced reactive astrocytes (e.g., VCAM1, BST2, ICOSL, HLAE, PD-L1, PDPN), which have been proposed as potential indicators of this reactive state and possible signature of neurodegenerative diseases (Labib et al., 2022). Astrocytes overexpressing PP enzymes change some features of a fully homeostatic phenotype, as shown by alterations in mTOR signaling, cytoskeletal organization, and amino acid transporters. Decreasing mTOR activity in astrocytes can lead to a reduction in their proliferation, migration, and inflammatory mediator production. This is particularly relevant in the context of neurological conditions where astrocyte activation and reactivity contribute to disease pathology (Hochmuth and Hirrlinger, 2024; Zhang et al., 2025). Altogether, our data suggest that the changes we observe upon the overexpression of PHGDH/PP enzymes reflect a metabolic reprogramming rather than a transition to a classical reactive phenotype.

A significant picture is the upregulation of nucleotide metabolism following PHGDH/PP enzyme overexpression. Given the critical roles of purines and pyrimidines in brain development—and the association between their dysregulation and the onset of neurodegenerative diseases (Fumagalli et al., 2017; Kundu and Dubey, 2021)—this metabolic feature is of particular interest. Previous studies have shown that PHGDH supports nucleotide biosynthesis by fueling both the pentose phosphate pathway and the TCA cycle, thereby sustaining anabolic demands in proliferating tumor cells (Reid et al., 2018). Here, we show a similar mechanism in differentiated human astrocytes, providing additional examples of conserved metabolic regulatory mechanisms, concerning also the other two enzymes of the PP.

In conclusion, peculiar alterations are apparent when each enzyme of the PP is overexpressed, strongly supporting the use of hiPSC-derived astrocytes overexpressing the PP enzymes as a valuable cellular model for understanding Ser metabolism. Future studies could also explore the signaling interactions among neurons and glial cells, potentially opening new therapeutic options for various brain diseases.

## Data Availability

The datasets presented in this study can be found in online repositories. The names of the repository/repositories and accession number(s) can be found below: https://www.ebi.ac.uk/pride/archive/, PXD045073.

## Footnotes

1
https://imagej.net/software/fiji/

2
www.biochem.mpg.de/mann/tools/
