# Supplementary material for: Modulating the serine metabolism in human differentiated astrocytes: an integrated multi omics approach
Source: Front Cell Neurosci. 2025 Jul 21;19:1616911. doi: 10.3389/fncel.2025.1616911 (PMC12318971; doi:10.3389/fncel.2025.1616911)
Supplement: Supplementary file 6 [file Data_Sheet_1.docx]

***Supplementary Material***

# Supplementary Figures and Tables

## Supplementary FiguresFigure S1
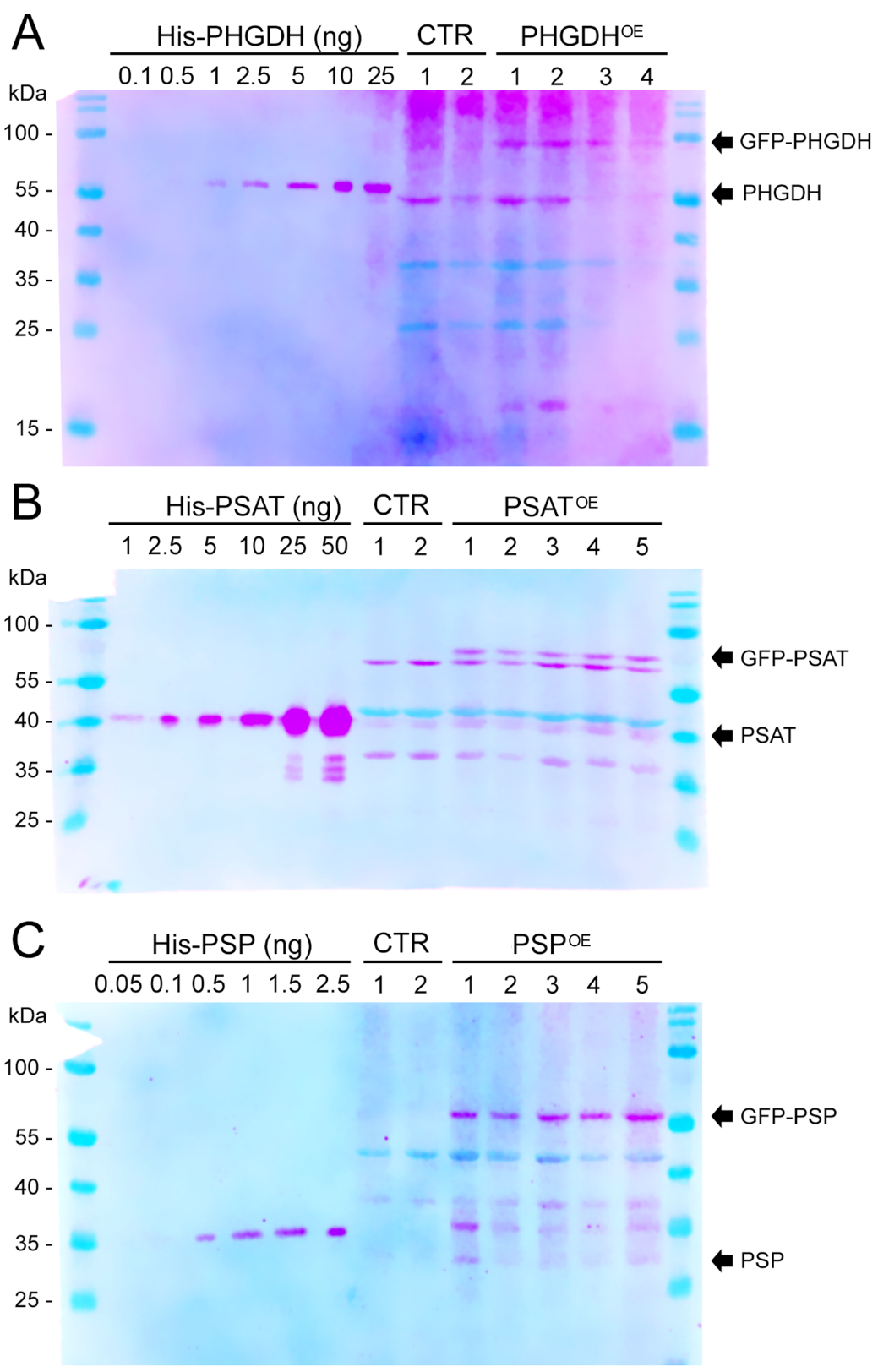


**Figure S1.** Representative Western blot analysis for the determination of the expression levels of PHGDH (A), PSAT (B) and PSP (C) in hiPSC-derived astrocytes over-expressing the corresponding protein. The signal recognized using primary antibodies against PHGDH, PSAT or PSP is shown in magenta; the signal detected using the anti-GAPDH antibody, used for the signal normalization, is shown in light blue. The arrows indicate the endogenous or GFP-tagged full-length enzymes. 20 μg of total proteins were loaded in each lane. The recombinant standard proteins show a slightly lower electrophoretic mobility due to the presence of an additional His-tag.

**Figure S2**

**
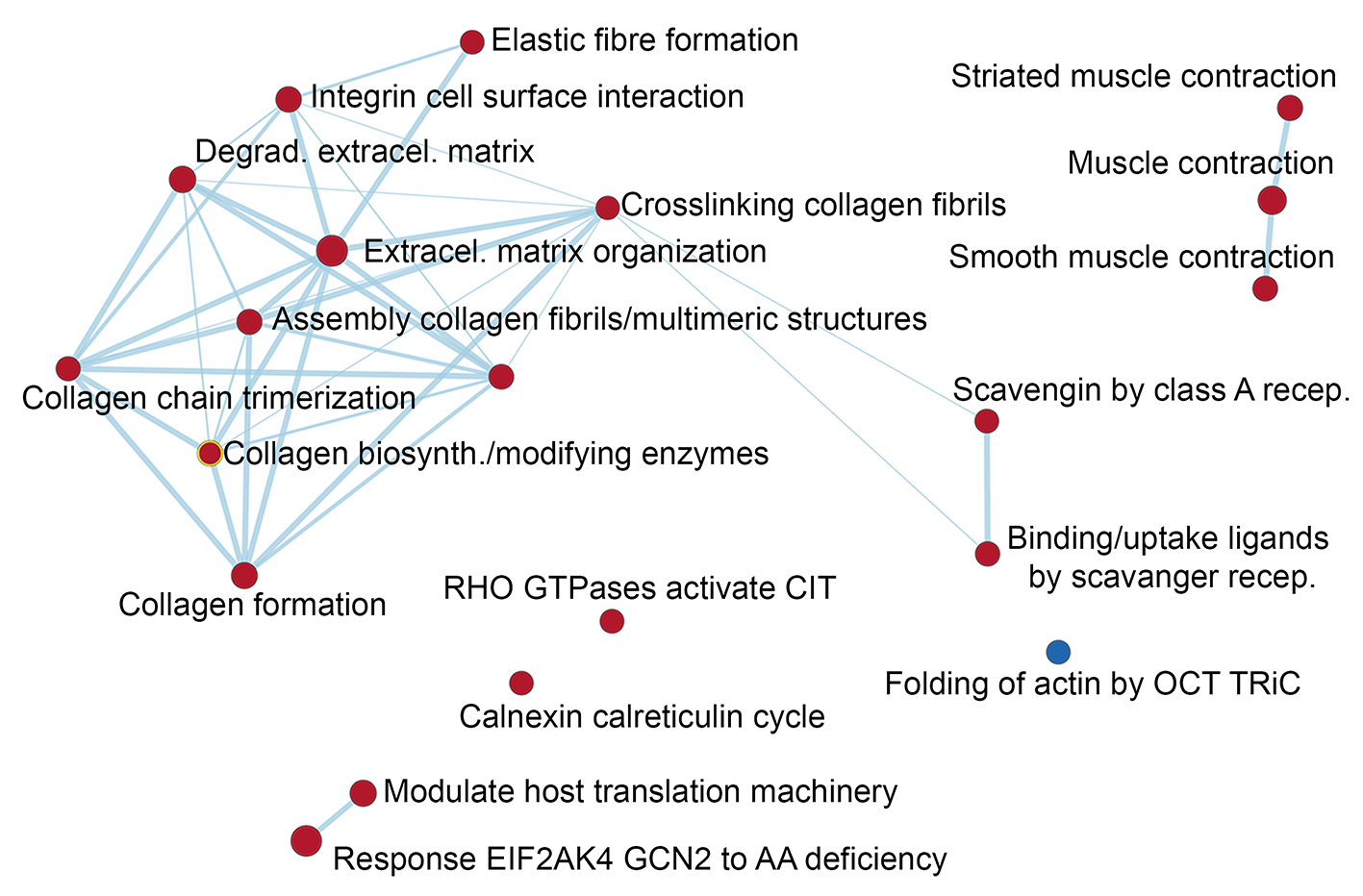
**

**Figure S2.** Enrichment map upon GSEA analysis of the proteins differentially expressed in the CTR vs non-transduced mature astrocytes comparison. The proteins differentially expressed in the comparison CTR vs non-transduced mature astrocytes were analyzed by GSEA to find enrichment in reactome pathways and a network representing overlaps among enriched pathways was created by Cluego - Enrichment Map. In red and blue the pathways increased or decreased, respectively. Node size represents the number of genes in the gene-set; edge thickness is proportional to the overlap between gene-sets, calculated using the Jaccard or overlap coefficients.

**Figure S3**

**
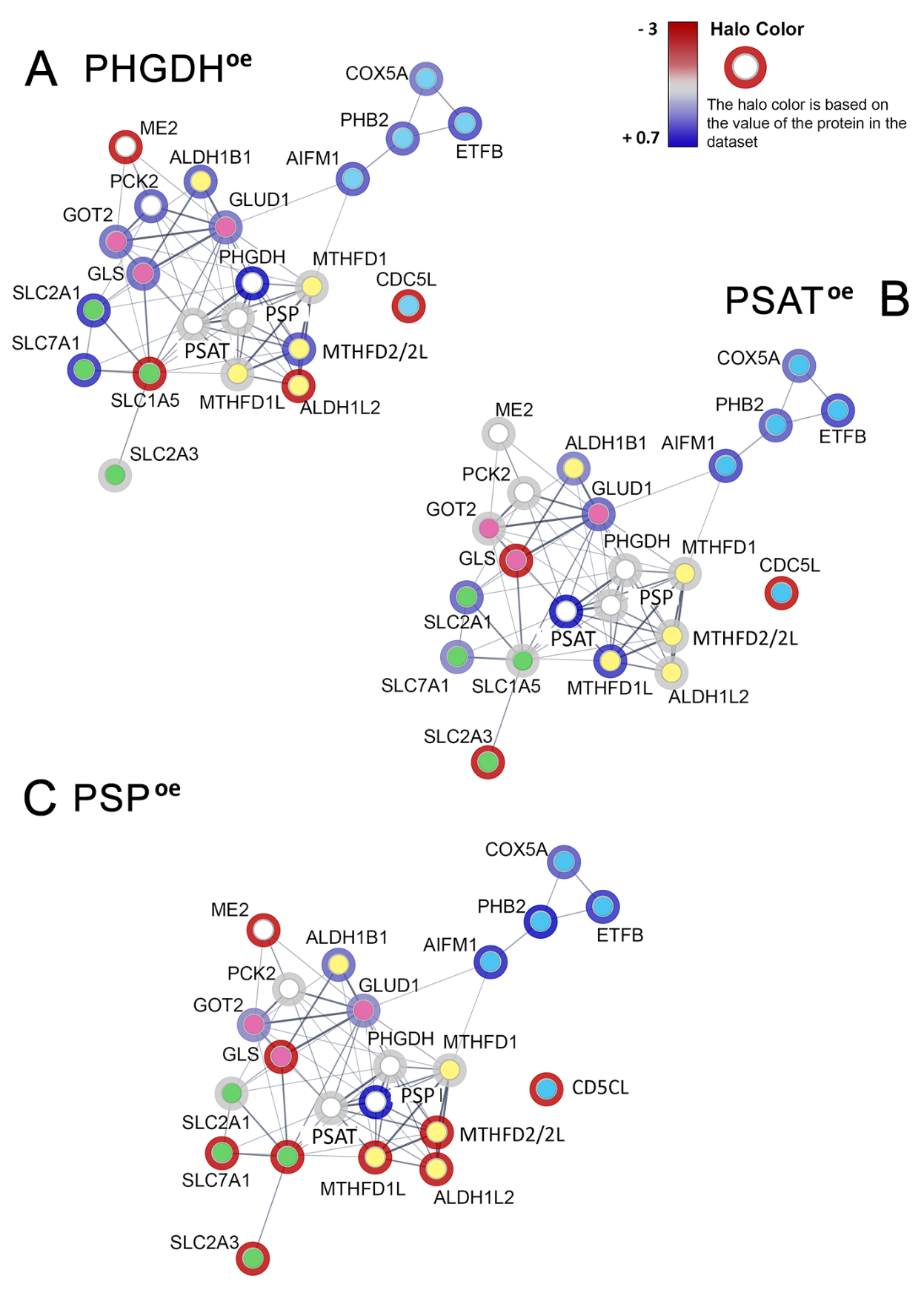
**

**Figure S3.** Summary by String of the main proteins discussed in PHGDH^oe^, PSAT^oe^ and PSP^oe^ proteomics results. The main proteins discussed in the proteomic results in PHGDH^oe^ (A), PSAT^oe^ (B) and PSP^oe^ (C) were analyzed by String. The halo color is based on the intensity of the protein in the data sets from red (decreased) to blue (increased) in the following comparisons (A) PHGDH^oe^ vs CTR, (B) PSAT^oe^ vs CTR, and (C) PSP^oe^ vs CTR. The proteins whose expression changes are not statistically significant in the comparisons are indicated in gray. The expression levels of PHGDH, PSAT and PSP refer to the intensity measured by mass spectrometry in the proteomic analysis. In green: amino acids and glucose transporters; in magenta: Glu/Gln metabolism; in yellow: one C metabolism; in light blue: direct PGDH interactors. SLC1A5 = ASCT2, SLC7A1 = CAT1, SLC2A1 = GLUT1, SLC2A3 = GLUT3. The proteins are indicated by the official gene code. ME2, malic enzyme; PCK2, phosphoenolpyruvate carboxykinase [GTP], mitochondrial; GOT2, aspartate aminotransferase, mitochondrial; GLS, glutaminase kidney isoform, mitochondrial; SLC2A1, solute carrier family 2, facilitated glucose transporter member 1; SLC7A1, high affinity cationic amino acid transporter 1; SLC1A5, neutral amino acid transporter B(0); SLC2A3, solute carrier family 2, facilitated glucose transporter member 3; MTHFD 1/1L, methylenetetrahydrofolate dehydrogenase 1/1L mitochondrial; PHGDH, D-3-phosphoglycerate dehydrogenase; PSAT, phosphoserine aminotransferase; PSP, phosphoserine phosphatase ALDH1L2, aldehyde dehydrogenase 1 family member L2 mitochondrial; MTHFD2/2L, methylenetetrahydrofolate dehydrogenase 2/2L mitochondrial; ALDH1B1, aldehyde dehydrogenase mitochondrial; GLUD1, glutamate dehydrogenase 1, mitochondrial; AIFM1, apoptosis inducing factor mitochondria associated 1; PHB2, prohibitin-2; COX5A, cytochrome c oxidase subunit 5A, mitochondrial; ETFB, electron transfer flavoprotein subunit beta; CDC5L, cell division cycle 5-like protein.

**Figure S4**


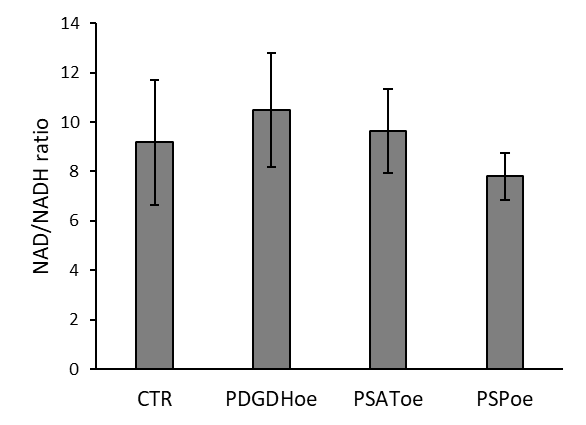


**Figure S4.** NAD^+^/NADH ratio in astrocytes overexpressing PHGDH, PSAT and PSP.

## Supplementary Table legends

**Table S1.** Quantification of the identified metabolites.

**Table S2-S4.** Proteins differently or exclusively expressed in the comparison PHGDH^oe^ vs CTR (S2), PSAT^oe^ vs CTR (S3), PSP^oe^ vs CTR (S4). Proteins were considered differentially expressed in the comparison if they showed significant t-test difference (Student’s t-test FDR ≤0.05) or are expressed exclusively in one condition.

**Table S5.** Bioinformatic analysis by Panther of the proteins differentially expressed in the comparisons PHGDH^oe^ vs CTR, PSAT^oe^ vs CTR and PSP^oe^ vs CTR. The analysis was conducted by Panther for Biological Processes, Molecular Function and Pathways enrichment. Functional grouping was based on p-value ≤ 0.05 and at least two counts.

**Table S6.** Bioinformatic analysis by IPA of the proteins differentially expressed in the comparison PHGDH^oe^ vs CTR, PSAT^oe^ vs CTR, PSP^oe^ vs CTR. The analysis was conducted by IPA on the data sets reported in Tables S3-S5. For each data set, the table lists the pathways with an activation Z score and the involved proteins.

**Table S7.** Proteins differently or exclusively expressed in the comparison CTR vs 30 days of differentiation (non-transduced astrocytes). Proteins were considered differentially expressed in the comparison if they showed significant t-test difference (Student’s t-test FDR ≤0.05) or are expressed exclusively in one condition.
